# Application of 3D printing in assessment and demonstration of stab injuries

**DOI:** 10.1007/s00414-022-02846-6

**Published:** 2022-06-03

**Authors:** Gábor Simon, Dénes Tóth, Veronika Heckmann, Viktor Soma Poór

**Affiliations:** Department of Forensic Medicine, University of Pécs Medical School, Szigeti út 12, 7624 Pécs, Hungary

**Keywords:** Forensic pathology, Autopsy, Stab wound, 3D printing, Photogrammetry

## Abstract

In stabbing related fatalities, the forensic pathologist has to assess the direction of wound track (thus, the direction of the stabbing) and the weapon’s possible characteristics by examining the stab wound. The determination of these characteristics can be made only with a high level of uncertainty, and the precise direction of the stabbing is often difficult to assess if only soft tissues are injured. Previously reported techniques used for the assessment of these wound characteristics have substantial limitations. This manuscript presents a method using today’s easily accessible three-dimensional (3D) printing technology for blade-wound comparison and wound track determination. Scanning and 3D printing of knives is a useful method to identify weapons and determine the precise stabbing direction in a stabbing incident without compromising the trace evidence or the autopsy results. Ballistic gel experiment, and dynamic stabbing test experiments prove the method can be applied in safety, without compromising the autopsy results. Identification of the exact knife is not possible with complete certainty but excluding certain knives will decrease the number of necessary DNA examinations, hence it can lower the burden on forensic genetic laboratories. The method addresses many of the shortcomings of previously used methods of probe insertion or post-mortem CT. Insertion of the printed knife into the wound gives a good visual demonstration of the stabbing direction, thus easing the forensic reconstruction of the stabbing incident. After combining the 3D printing with photogrammetry, the achieved 3D visualization is useful for courtroom demonstration and educational purposes.

**Supplementary Information:**

The online version contains supplementary material available at 10.1007/s00414-022-02846-6.

## Introduction

In stabbing related fatalities, the forensic pathologist must assess the direction of wound track (thus, the direction of the stabbing), and the possible characteristics of the weapon by examining the stab wound. These characteristics include the length, width, and thickness of the blade, number and type of edges, and the degree of taper from tip to hilt [1]. The determination of these characteristics can be made only with a high level of uncertainty. The length of the stab lesions may suggest the width of the knife blade, but the skin may retract slightly because of its elasticity, which would decrease the size of the wound, thus hampering the identification of the knife [2]. The number and the type of edges are the most easily assessed characteristics, but Amadasi et al. reported a success rate of only 48.0% overall [3].

If a knife linked to the case is found, the forensic pathologist must assess whether it could have created the wound. Knives possibly used in stabbing should be taken to the autopsy to compare the blade with the stab wound [2]. In the case of multiple knives found, sorting out the most likely weapon can be a challenging task. The exclusion of some of the weapons may be possible, but it is usually not possible to link definitely a knife to a wound unless broken parts of the blade are embedded into the body [4]. There are some wound-blade comparison techniques described in the literature, but all of them have substantial limitations [5–7].

The precise direction of the stabbing has to be assessed by careful dissection of the tissues layer by layers starting from the surface downward. It can be determined with a great degree of probability if two bony structures have been struck along the track, but it is more difficult if only soft tissues are injured [2]. A post-mortem computer tomography (PMCT) is a valuable tool for this task, but not cost-effective and has limitations as discussed below.

Three-dimensional (3D) printing or additive manufacturing revolutionized several fields of technology and science in recent years, allowing for rapid prototyping and preparation of unique objects. The rapid decrease of costs makes this technology more accessible; the most affordable 3D printers are approximately $200 (US) at the moment of writing this manuscript. The most common technology is fused deposition modelling (FDM), where the printer melts a continuous filament of thermoplastic material and deposits it in precise layers. Several filament materials are available for 3D printing; we have chosen polylactic acid (PLA) for this test because it is mechanically strong, chemically resilient, and easy to print. 3D printing is increasingly used in medicine [8] and in forensics during evidence reconstruction and courtroom presentation [9–11], but no previous study report was found using it to recreate blades for wound-weapon comparison.

In this manuscript, we present a method using today’s easily accessible 3D printing for blade-wound comparison and wound track determination, developed to overcome some of the limitations of previous accessible methods. With the use of photogrammetry, this method can also give a suggestive demonstration of the stab wounds.

## Materials and methods

### Scanning and printing

Knives were scanned together with a ruler by a slightly modified Canon Canoscan LiDE 25 (Canon, Japan) flatbed scanner. A portion of the scanner’s side was removed, which allowed us to place the blades flat on the scanning glass. The scanner was decontaminated with DNA AWAY™ solution (Thermo Fisher Scientific, USA), but it is always advised to perform sampling for DNA analysis prior the scanning step. In this study, the 300 dpi resolution images were saved as tagged image file format (TIFF). Scanning with 600 dpi can also be recommended, since it makes the outline tracing a bit easier. The thickness of the blade was measured with an electronic outside micrometer (Helios-Preisser, Germany).

**Fig. 1:**
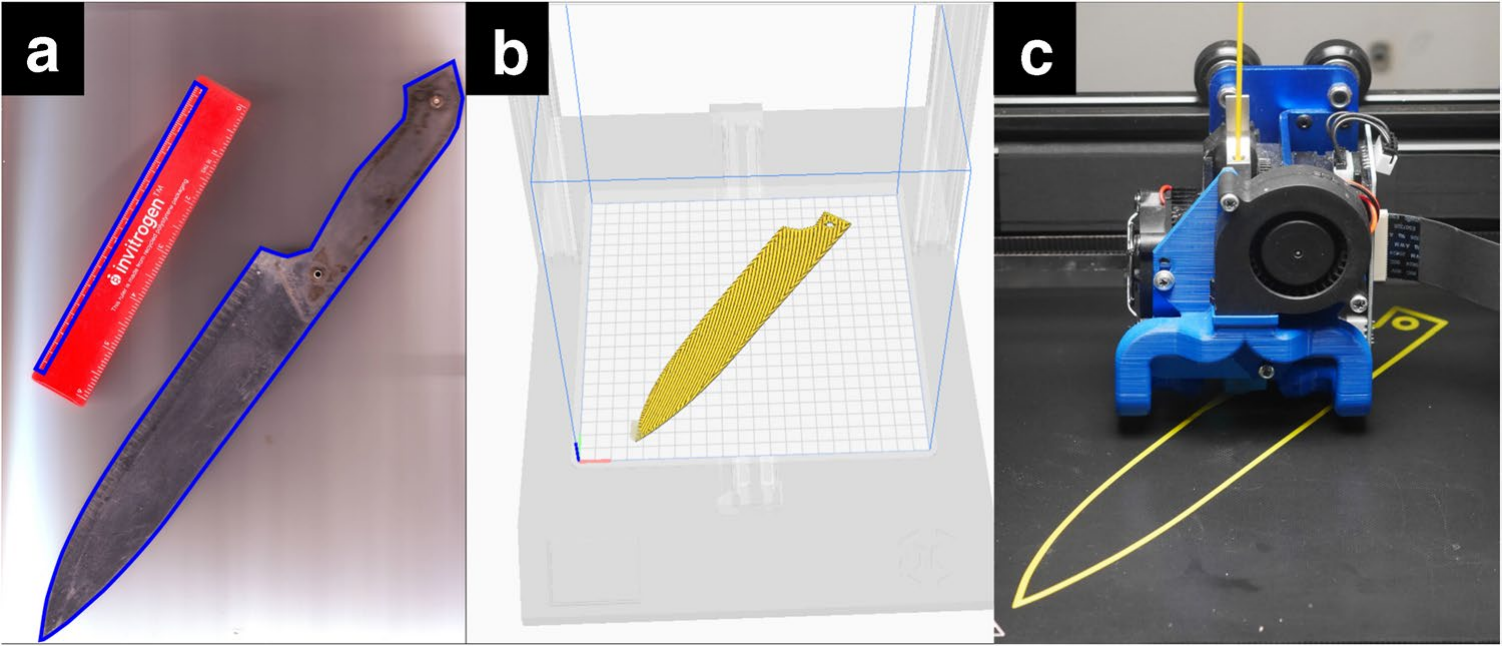
Scanning and 3D printing of the knives. Knives were scanned together with a ruler by a flatbed scanner. Contours of the blades were traced in Inkscape (**a**). The models were sliced for 3D printing in Ultimaker Cura (**b**). The objects were printed with an Artillery Genius 3D printer (**c**)

Images were opened in Inkscape™: an open-source scalable vector graphics editor (inkscape.org), and the contours of the blades were manually drawn with the Bezier curve and line tool (Fig. 1a). The path was exported as openSCAD (.scad) file and imported directly to Cura (Ultimaker B.V., Netherlands) slicer software (Fig. 1b) which processes the 3D models for 3D printing. The edge of the blades was not modelled to avoid possible tissue damage (the thickness of models was even). First the ruler was imported, its size was checked in the slicer software. If it was any different to the known size, the necessary scaling factor was calculated: (known size / size in slicer) * 100. The knife was scaled accordingly.

The scanning and editing steps were completed in about 20 min. We have provided a detailed tutorial as a supplementary file describing all the installation and editing steps (SI 1).

Printing parameters were nozzle width: 0.4 mm, printing temperature: 200 °C, bed temperature: 60 °C, layer height: 0.2 mm, infill: 100%, and printing speed: 50 mm/s. The knives were printed from PLA, polylactic acid (Filanora.eu, Hungary) with an Artillery Genius 3D printer (Shenzhen Yuntuchuangzhi Technology, China) in approximately 60 min each (Fig. 1c). Thickness of the printed blades was compared with the original knives with an electronic outside micrometer (Helios-Preisser, Germany), and other dimensions were assessed with a Vernier caliper (Mitutoyo, Japan). The maximal deviation was 0.4 mm on the horizontal plane (*X* and *Y* axes, length and width of blade), and it was 0.05 mm in thickness (vertical, *Z* axis).

### Ballistic gel experiment

Ballistic gel block was created (Defensible Ballistics, 10% gel powder, 90% water) for the free-hand stabbing tests. The original knife labelled 1 was stabbed into the ballistic gel. A marking at the maximal penetration depth was placed on the blade. After removal of the original blade, its 3D printed replica was inserted into the wound and pressed down with considerable force. A marking was placed on the replica blade at the achieved maximal penetration depth. Markings on the original and replica blades were compared.

### Free-hand pork loin stabbing experiment

The original blade was stabbed into a pork loin, then the entrance wound length was measured by a digital caliper. Replica knives were installed into the same stab wounds, and maximal possible horizontal force was applied in the direction of its edge (horizontal to the skin surface). Entrance wound length was measured again by a digital caliper.

### Dynamic stabbing force measurements

**Fig. 2:**
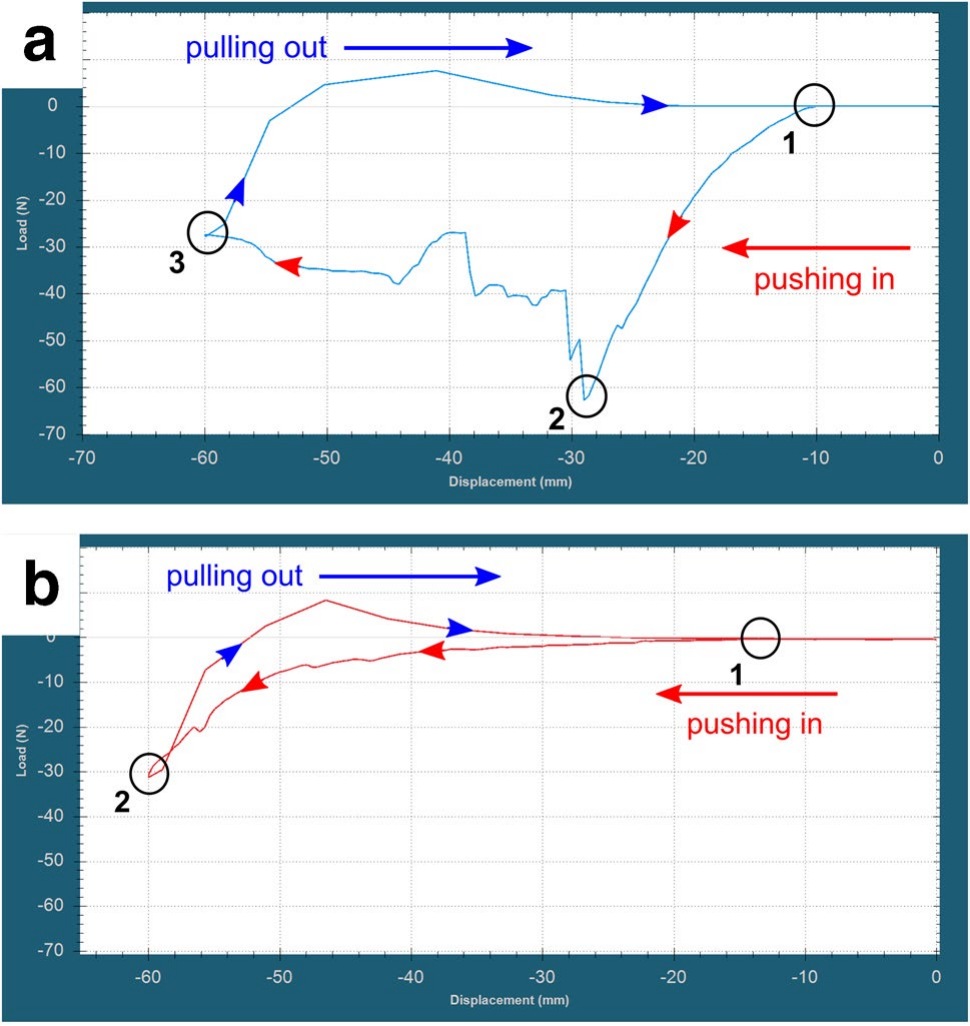
Explanation of the registered curves during dynamic force measurements: creation of the stab wound with original knife (**a**) and stabbing the plastic replica knife into the stab wound (**b**). Red arrows (“pushing in”) indicating the downward movement (stabbing), blue arrows (“pulling out”) indicating the upward movement of the blade. Number (1) indicates where tip of the blade reaches the surface of the pork loin, number (2) indicates the maximum force, while number (3) indicates the maximum displacement — note that the last two is the same in case of plastic replica knife

Dynamic stabbing force measurements were performed with a Mecmesin MultiTest-dv motorized test stand (2.5 kN) combined with a Mecmesin force gauge AFG-500 (0–500 N capacity, ± 0.5 N accuracy). Stabbing force was 2.5 kN (250 kg), initial tip–surface distance was 10 mm, and displacement (downwards movement) was 60 and 75 mm, with a constant movement speed of 100 mm/min. The load (force) was measured by AFG-500 was recorded with Mecmesin VectorPro Software (Fig. 2). Wound size was measured with a digital caliper. After creating a stab wound with the original blade on the pork loin, the replica blade was into the stab wound previously created by the original blade. A larger replica blade (label 2) was also stabbed into the same wound. The tests were performed again in such a way that the replica knives were stabbed 15 mm further than the original knife (75 mm overall displacement).

### Model experiment for wound assessment

**Fig. 3:**
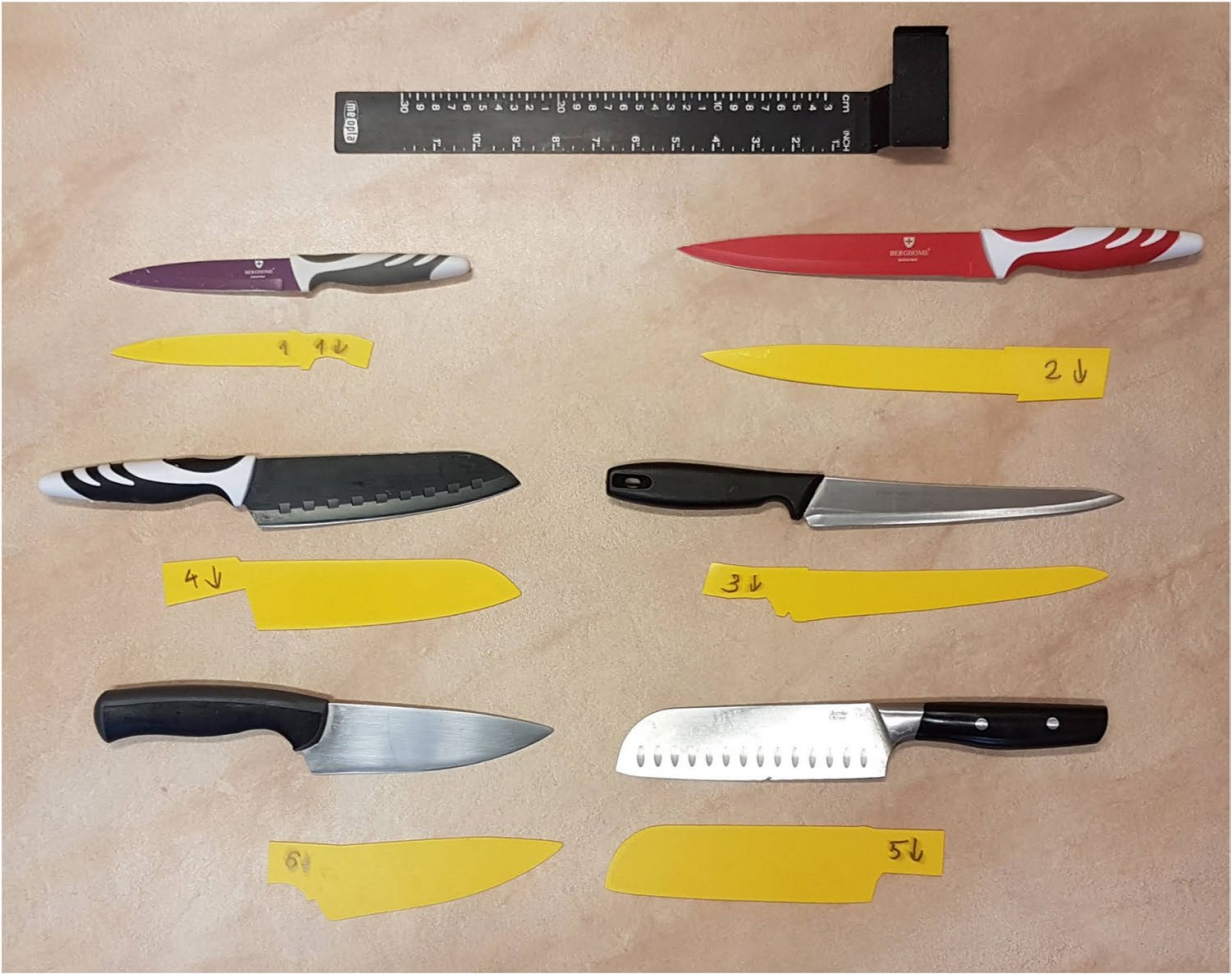
The original and printed knives

For the model experiment, six ordinary kitchen knives, labelled from 1 to 6 (Table 1), were scanned and printed (Fig. 3). Bone-in pork loin with skin was purchased from a local butcher. Seven wounds were produced randomly with the knives described in Table 1. Five participants with various forensic pathology experience (Table 2) were asked to compare the wounds with the printed knives and place the knives into the following categories: probable, possible, excluded. The categorization “probable” and “possible” was determined by the subjective judgement of the participants. All participants received the following instruction: category probable should be used if the blade fits into the wound perfectly by the participant’s opinion; category “possible” should be used if installing the blade into the wound is possible to full length of the wound track, but the fit is not perfect (so the blade can be moved transversally to its longitudinal axis). If the blade could not cause the wound by the opinion of the examiner, category “excluded” was used (as a definite statement).

**Table 1:** Type and dimensions of the various knives

Knife no	Brand	Blade length (mm)	Max. width (mm)	Max. thickness (mm)	Tip angle (°)
1	Berghome	119	22	1.2	33
2	Berghome	194	28	1.9	34
3	Fiskars	195	30.5	1.5	36
4	Berghome	173	43	1.8	65
5	Jamie Oliver	165	46	2.0	79
6	Ikea	139	44	1.6	38

**Table 2:** Participants and their experience in forensic pathology

Participant no	Experience in forensic pathology
1	Undergraduate student, without learning forensic medicine course
2	Nine years of experience as a forensic pathologist
3	Four years of experience as a forensic pathologist
4	38 years of experience as a forensic pathologist
5	Five years of experience as a forensic pathologist resident

The experiment was randomized and blinded. The stabbing order of the knives was randomized with rolls of a dice. The participants did not know if all the knives were used, and they performed the analysis separately.

### Photogrammetry

Photographs were taken using Canon EOS 600D (18 megapixels) with EF-S 18–55 mm kit lens. Camera setup included aperture priority (AV) f stops set approximately 18 to 22 with ISO between 1600 and 3200 and white balance adjusted to the room light temperature. Natural window light and light fixtures above the autopsy table were able to illuminate the entire surface of the body. Photographs were taken handheld from different points of view and with an overlap area of about 60–70%. Three sets of pictures were taken of each object: one round from a low perspective, one round at approximately 45°, and one from above. Images were transferred to a computer, and the blurred and out of focus images were erased. The remaining image sets were processed with Autodesk ReCap Photo (AutoDesk Inc., USA). The models are shown as Supplementary videos.

### Statistical analysis

The summarized outcomes were compared with chi-square test using online GraphPad QuickCalcs (https://www.graphpad.com/quickcalcs).

## Results

### Free-hand stabbing experiments

**Fig. 4:**
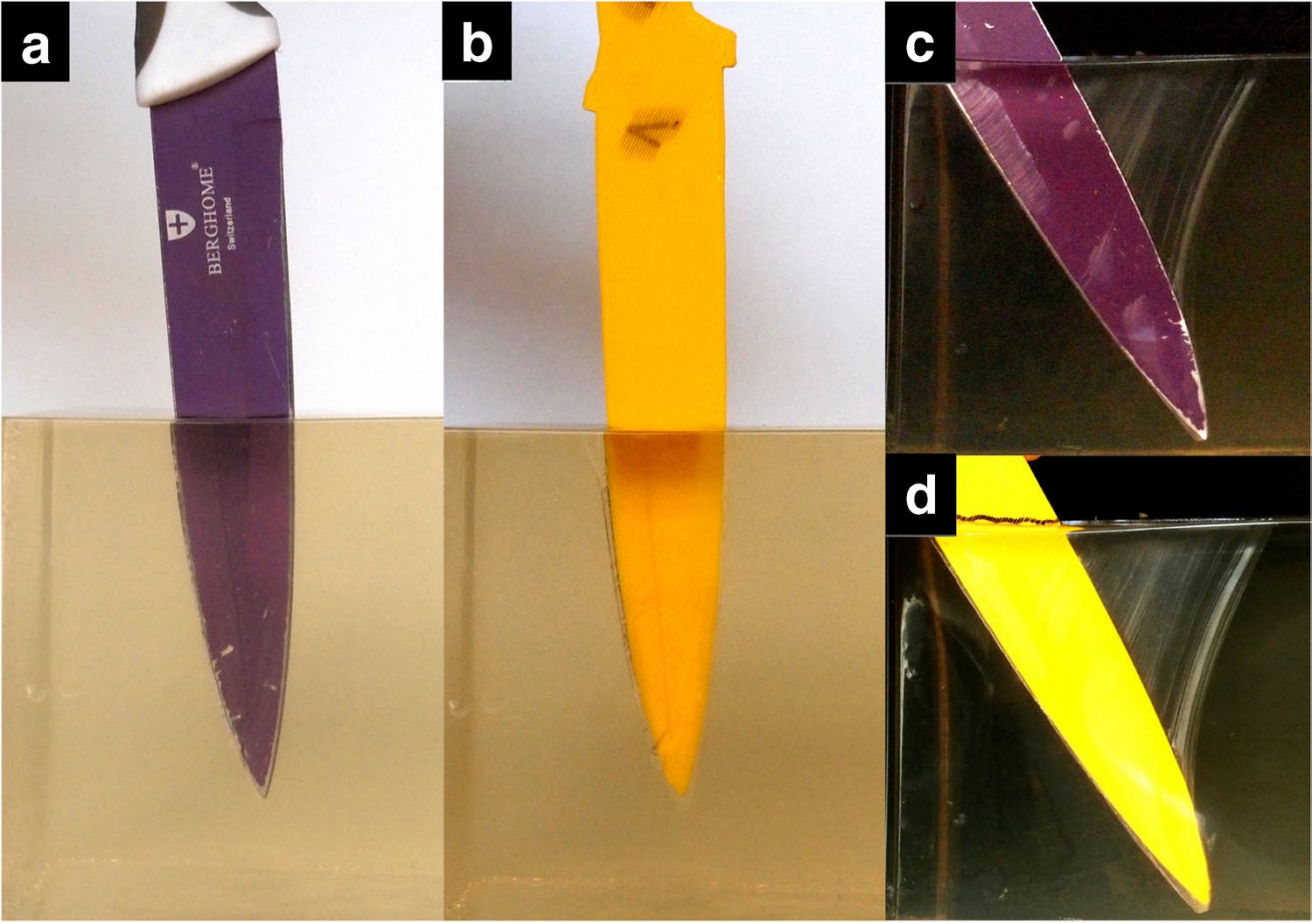
The stab wounds in ballistic gel. Stabbing with original blade (**a**, **c**), and insertion of replica blade into the stab wound (**b**, **d**)

The 3D printed version fit perfectly to the stab wound in the ballistic gel, and it was impossible to stab it deeper or enlarge the wound by applying considerable force (Fig. 4a–d). The place of markings on the original blade and on the replica matched.

### Free-hand pork loin stabbing experiment

By applying maximal horizontal force towards the pointed end of the entrance wound with the replica knife, entrance wound was enlarged by average 0.48 mm (0–0.61 mm) (Table 3).

**Table 3:** Entrance wound lengths in pork cutlet after applying maximal horizontal force towards the pointed end of the entrance wound with the replica knife

Stab no	Entrance wound length		Enlargement (%)
	Metal blade	Replica blade	
1	20.88 mm	21.17 mm	1.3
2	22.29 mm	22.29 mm	0
3	20.54 mm	21.15 mm	2.9
4	20.11 mm	20.32 mm	1
5	20.42 mm	20.81 mm	1.9

### Dynamic force measurements

**Fig. 5:**
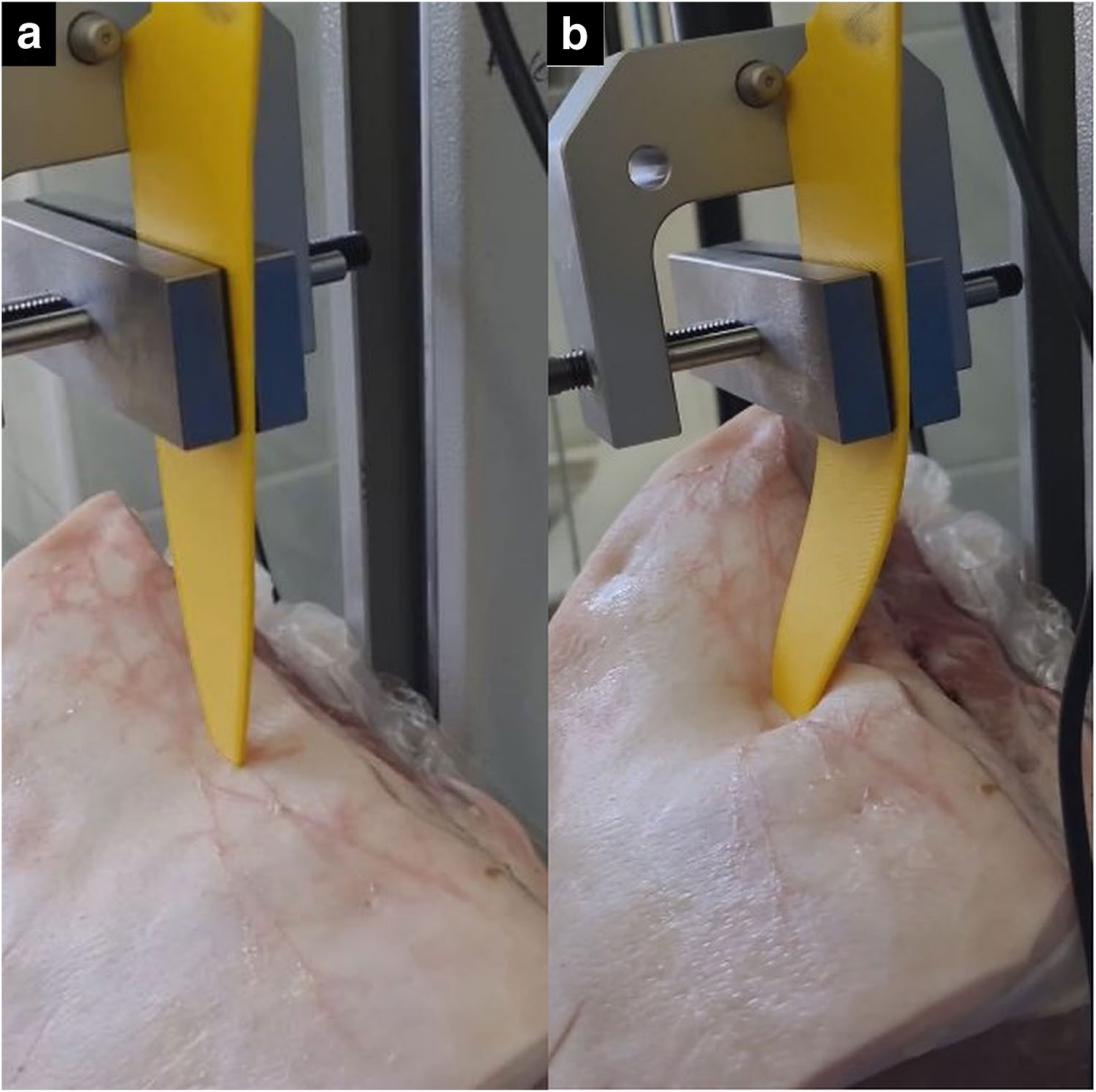
The oversized replica knife entering the stab wound (**a**), and it’s buckling as it reaches the borders of the entrance wound (**b**)

**Fig. 6:**
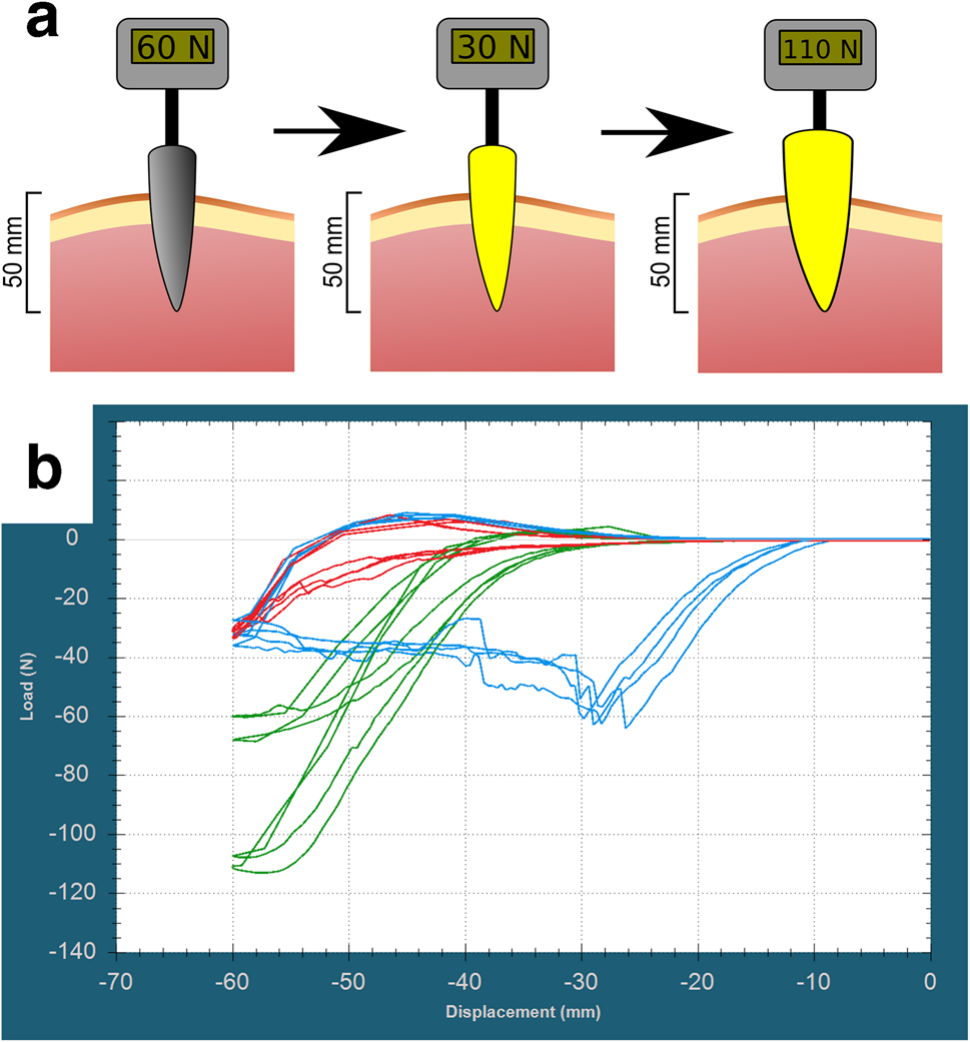
Explanatory illustration (**a**) and registered curves (**b**) of dynamic stabbing force with 60 mm total displacement (note that tip-surface distance was 10 mm). Original metal blade label 1 (blue), replica of the blade label 1 (red), replica of the blade label 2 (green)

The average maximum load during stabbing with original blade (label 1) was 63.3 N (62.2–64.3 N) with a constant load between 30 and 40 N after penetration. Average load was 32.1 N (27.4–36 N) at 60 mm displacement. Stab wound depth was 34–36 mm. The load during stabbing the replica blade into the previous stab wound with was between 1 and 5 N (average 3.8 N at 40 mm displacement). The load started to rise rapidly as the blade reached the previously uninjured tissues and reached an average load of 32.88 N (31.5–33.9 N) at 60 mm displacement. The load during stabbing with the larger replica blade (label 2) started to increase rapidly as it reached the edges of the entrance wound, and the blade also started to buckle (Fig. 5, SI 2). The average load at 40 mm displacement was 20.2 N (14.7–2.9), and the maximum load was 87.75 N (62.5–113.2 N) (Fig. 6). Table 4 shows the length of the entrance wounds.

**Table 4:** Entrance wound length of stab wound in pork cutlet at 60 mm displacement

Wound no	Metal blade (label 1)	Replica blade (label 1)	Replica blade (label 2)
1	14.75 mm	14.75 mm	16.02 mm
2	14.38 mm	14.52 mm	15.63 mm
3	14.65 mm	14.66 mm	15.57 mm
4	14.66 mm	14.90 mm	15.80 mm

**Fig. 7:**
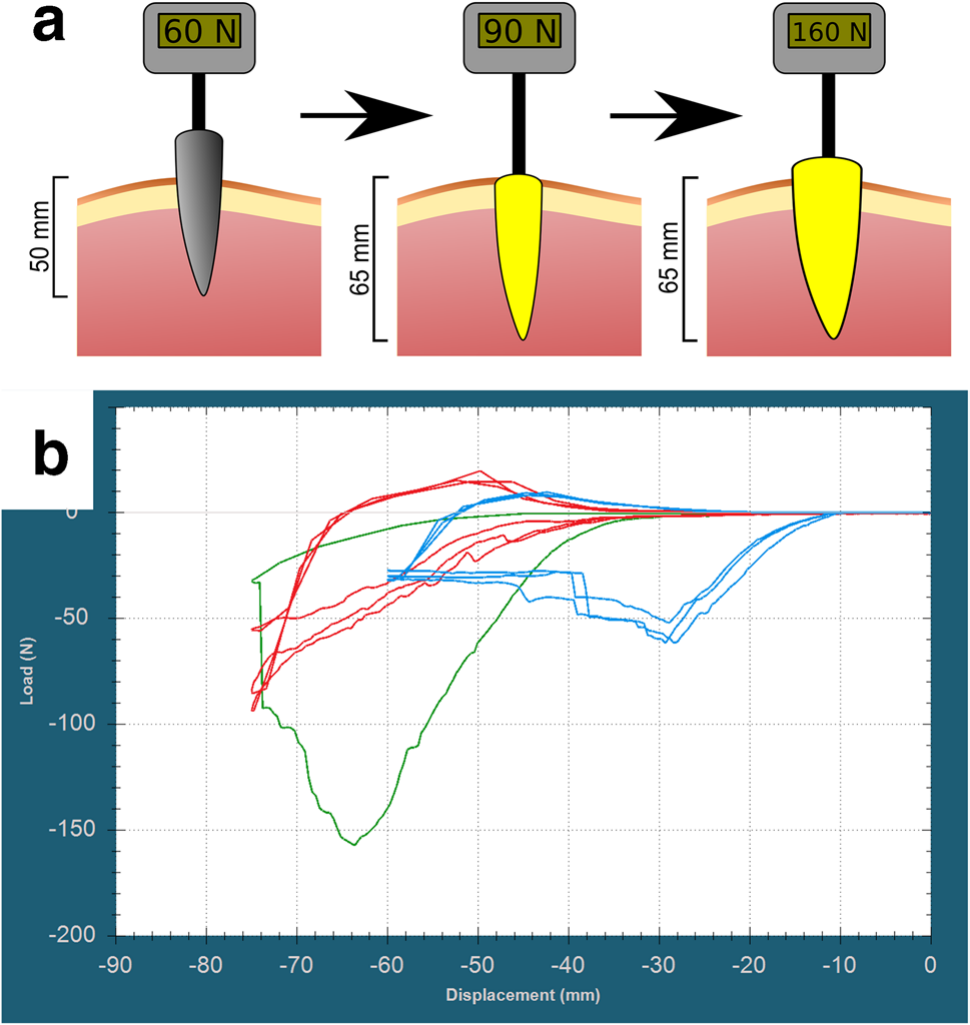
Explanatory illustration (**a**) and registered curves (**b**) of dynamic stabbing force measurements with 60 mm total displacement of original, and 75 mm total displacement of replica blades (note that tip-surface distance was 10 mm). Original metal blade label 1 (blue), replica of the blade label 1 (red), replica of the blade label 2 (green)

In the second series of measurements, average maximum load with stabbing with original blade (label 1) was 59.3 N (52.9–62.6 N) with a constant load between 30 and 40 N after penetration. Average maximum load was 38.7 N (33.9–44.2) at 60 mm displacement. Stab wound depth was 33–34 mm. An average load during stabbing the replica blade into the previous stab wound with was between 1 and 5 N (average 5.5 N at 40 mm displacement). The load started to rise rapidly as the blade reached the previously uninjured tissues and a slight buckling was observed after 60 mm displacement. Average load was 37.95 N (33.9–44.2 N) at 60 mm blade displacement, and average maximal load at 75 mm displacement was 78.8 N (56.9–94.2 N). The load during stabbing with the larger replica blade (label 2) started to increase rapidly as it reached the edges of the entrance wound, and the blade also started to severely buckle and fracturing at 63.3 mm displacement (total fracture occurred at 74 mm displacement). The load was 140.8 N at 60 mm displacement and maximum load was 157 N at 63.63 mm displacement (Fig. 7). Table 5 shows the length of the entrance wounds.

**Table 5:** Entrance wound length of stab wound in pork cutlet. Displacement is 60 mm in case of metal blade, and 75 mm in case of replica blades (note: replica blade label 2 fractured during the first measurement)

Wound no	Metal blade (label 1)	Replica blade (label 1)	Replica blade (label 2)
1	14.91 mm	14.92 mm	17.61 mm
2	14.60 mm	15.28 mm	
3	14.48 mm	15.80 mm	

### Model experiment for wound assessment

The success rate of identifying the knives based on the wound track was analyzed: Table 6 shows whether the knife causing the wound was categorized as probable, possible, or excluded. A wound labelled “d” and “e” with a higher rate of miss was created by the knives having similar endings (tips), while the wound labelled “g” was superficial, only 2 cm deep. Summarizing the outcomes, the participants have chosen 23 times altogether that the used knife was the most probable weapon and they 12 times the used knife as a possible candidate. None of the experts incorrectly excluded the used knife. The differences between these groups were statistically significant (*p* < 0.0001). Table 7 shows how successful the categorization was in the case of different knives. The differences in success rate between different participants in linking the wounds with the knives can be found in Table 8.

**Table 6:** Success of linking knives with the stab wounds

Wound	Knife no	Probable	Possible	Incorrectly excluded
a	6	5 (100%)	0 (0%)	0 (0%)
b	4	4 (80%)	1 (20%)	0 (0%)
c	1	4 (80%)	1 (20%)	0 (0%)
d	1	1 (20%)	4 (80%)	0 (0%)
e	2	2 (40%)	3 (60%)	0 (0%)
f	3	4 (80%)	1 (20%)	0 (0%)
g	5	3 (60%)	2 (40%)	0 (0%)
Sum		23	12	0

**Table 7:** Success rate of identifying which wounds were caused by the particular knives

Knife	Probable	Possible	Incorrectly excluded
1	5 (50%)	5 (50%)	0 (0%)
2	2 (40%)	3 (60%)	0 (0%)
3	4 (80%)	1 (20%)	0 (0%)
4	4 (80%)	1 (20%)	0 (0%)
5	3 (60%)	2 (40%)	0 (0%)
6	5 (100%)	0 (0%)	0 (0%)

**Table 8:** Success rate of participants linking the wounds with the knives by the different participants

Participant	Probable	Possible	Incorrectly excluded
1	4 (57%)	3 (43%)	0 (0%)
2	6 (86%)	1 (14%)	0 (0%)
3	4 (57%)	3 (43%)	0 (0%)
4	2 (28%)	5 (71%)	0 (0%)
5	7 (100%)	0 (0%)	0 (0%)

Apart from blade length, knives nos. 1, 2, and 3 had similar characteristics (blade width/tip angle), so the results were examined if these three knives are handled as one. Results are shown in Table 9. When grouping the similar blades together, the participants identified the group containing the used knife 32 times as the most probable and 3 times the possible tool. There was no false exclusion. The difference between these groups proved to be statistically significant (*p* < 0.0001). In the case of the superficial wound labelled “g,” either knife no. 4 or 5 was picked as “probable.”

**Table 9:** Success of linking similar knives with the stab wounds

Wound	Knife no	Probable	Possible	Incorrectly excluded
a	6	5 (100%)	0 (0%)	0 (0%)
b	4	4 (80%)	1 (20%)	0 (0%)
c	1–2-3	5 (100%)	0 (0%)	0 (0%)
d	1–2-3	5 (100%)	0 (0%)	0 (0%)
e	1–2-3	5 (100%)	0 (0%)	0 (0%)
f	1–2-3	5 (100%)	0 (0%)	0 (0%)
g	5	3 (60%)	2 (60%)	0 (0%)
Sum		32	3	0

## Case demonstration

**Fig. 8:**
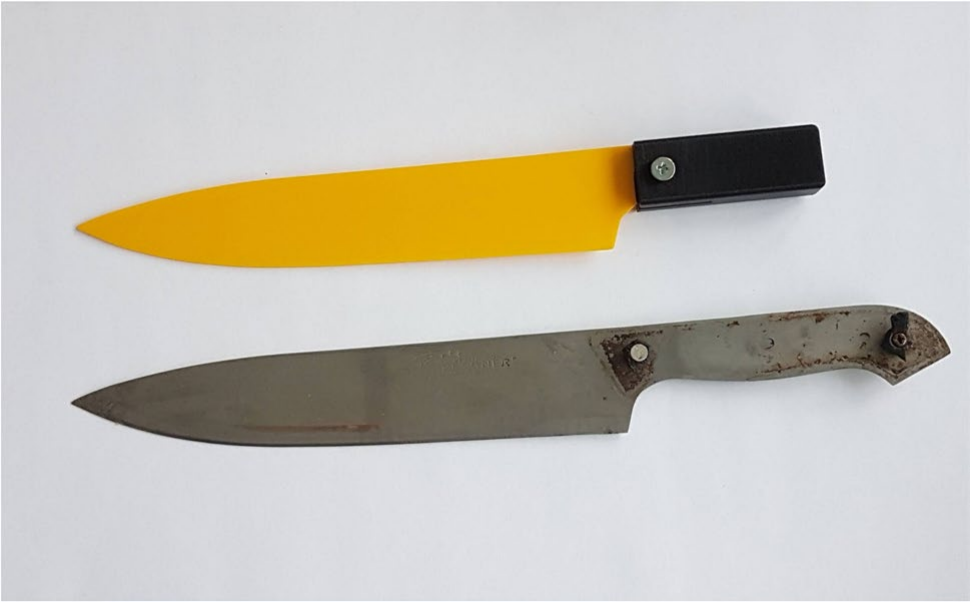
The original and 3D printed knife used in case demonstration

The method was put to the test in a real-life homicide case. A 20-year-old male was killed during a fight between four people. Prior to the autopsy, the knife found at the crime scene was handed over to the forensic pathologist for identification. A 3D printed model of the knife was created before the autopsy with the method discussed above (Fig. 8).

**Fig. 9:**
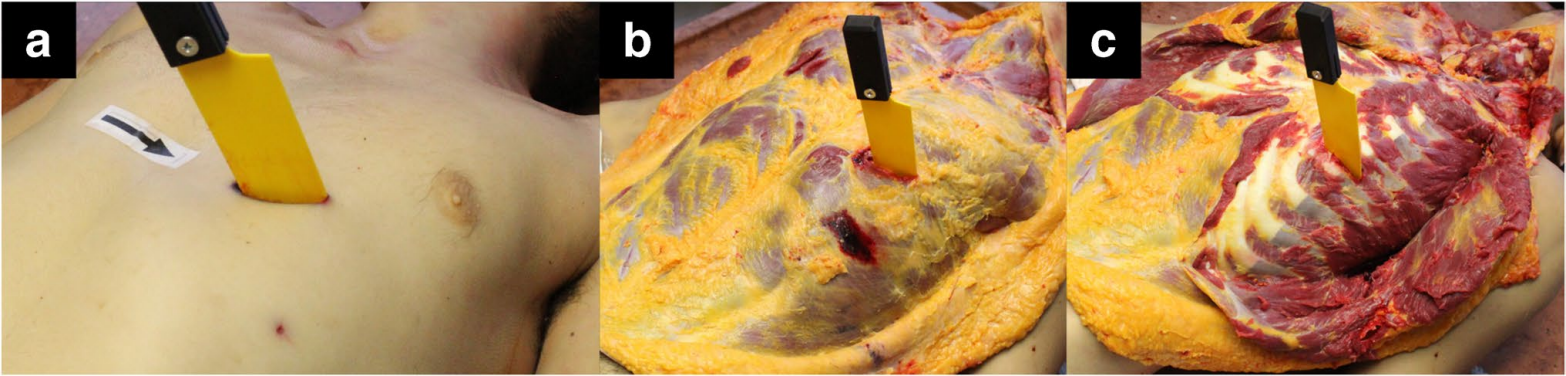
The stab wound in different layers

External examination revealed multiple injuries: multiple lacerations, contusions, and abrasions on the forehead, contusions and lacerations on the lips, an incision near the left eye, contusions in the mandibular region, small contusions and abrasion on the neck, several contusions and abrasions on the extremities, a puntiform lesion, and a large and a superficial stab wound on the chest. The stab wound was nearly horizontal, 39 mm long and 15 mm gaping. The medial end was clearly rounded, and the lateral was sharply pointed. The opened thoracic cavity could be observed at the base of the wound. After the conventional documentation methods, the printed knife was inserted into the wound, and photos were taken with the method discussed above to create a 3D model. The autopsy was continued with the usual layer-by-layer method following the wound track, the printed knife was inserted into each layer, and photos were taken for later 3D reconstruction (Fig. 9).

**Fig. 10:**
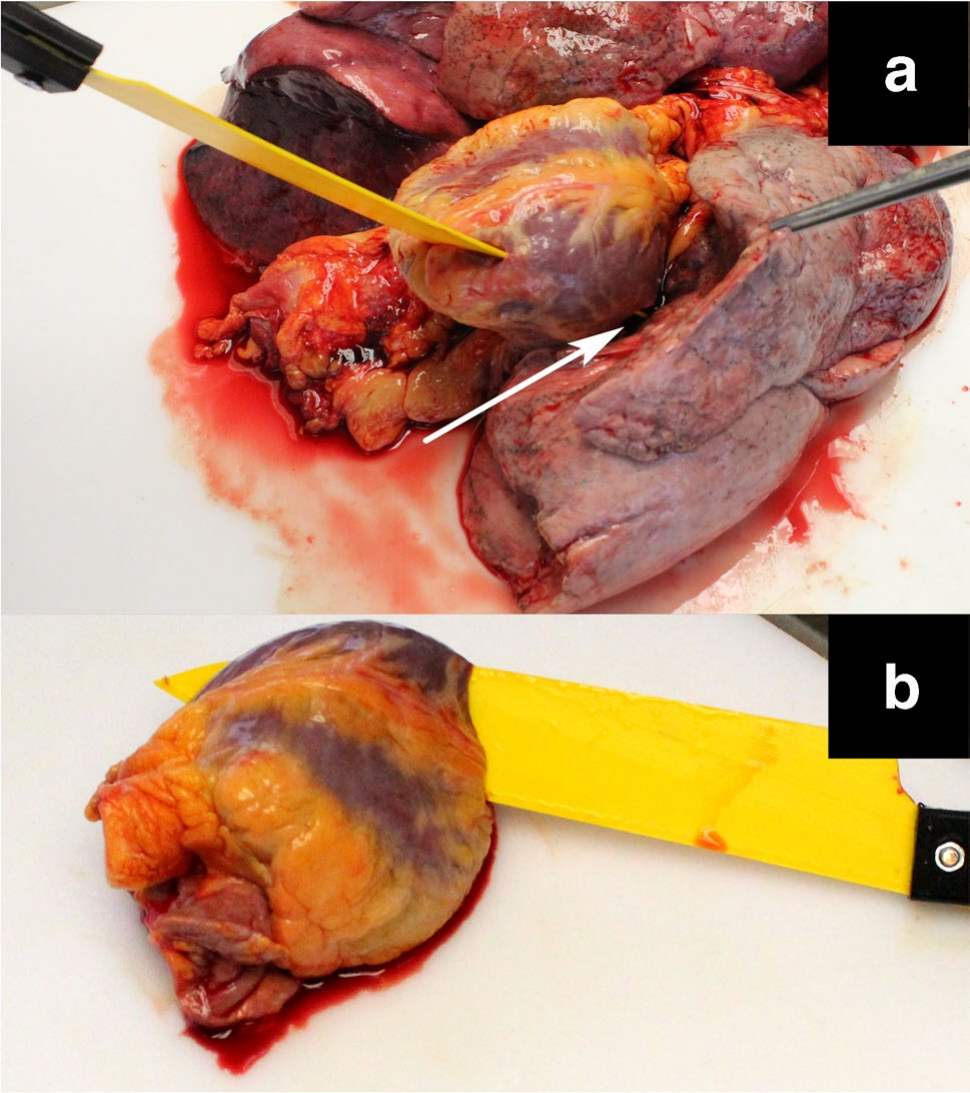
Injuries of the heart and lung

A near-horizontal stab wound was seen in the 5th intercostal space, with the medial end injuring the cartilage of the 6th rib. The thoracic cavity contained 3,200 ml of blood. On examination of the heart and lungs, a horizontal stab wound on the septum and anterior wall, a horizontal stab wound on the posterior wall of the left ventricle, and a horizontal stab wound on the lower left lobe of the lung near the hilus were found (Fig. 10). Apart from the stab wound, an autopsy revealed slight brain edema, minor bleeding in neck tissues, and shock kidneys.

The printed knife was inserted into the injuries of the heart–lung complex and photographed for later 3D reconstruction. 3D models were created using photogrammetry after the autopsy. Angle measurements were made based on the reconstructed 3D models using a Helios-Preisser protractor. The angle of the blade, thus the angle of the wound track, was 33° to the transverse, 15° to the sagittal, and 83° to the coronal plane. Based on the autopsy findings and the usage of the 3D printed knife and photogrammetric 3D reconstruction, it could be proven that the stabbing was directed slightly to the left and upwards with the lateral-looking cutting edge rotated slightly upwards. The blade went through the anterior and posterior walls of the left ventricle and ended up in the lung near the hilus. The cause of death was hemorrhagic shock. The 3D printed knife fitted perfectly into the wound track and allowed precise measurement of the wound track.

## Discussion

The most obvious method for blade identification would be inserting and fitting the particular weapon into the stab wound, but it cannot be advised for multiple reasons. The inserted (sharp) weapon can enlarge and deform the wound, thus limiting further assessment of autopsy findings. Even if the blade does not directly contact the body, the possibility of cross-contamination will always be there if the weapon is brought to the autopsy room [12].

Removing the tissues surrounding the stab wound for later comparison with the blade is a possible method [5], but it is not recommended since excision will cut through the elastic fibers, thus distorting the shape and size of the wound [7]. Furthermore, formalin fixation will result in shrinkage of the tissues making accurate and valid measurements impossible [7, 13].

Creating new stab wounds with a particular knife on a pigskin can be a useful method [6, 7]. Zohn et al. demonstrated that it is a reliable method used to determine the minimum possible wound length at an established depth of penetration [7]. The limitation of this method is that it does not give enough information about the weapon size if the blade movements enlarge the wound and does not help to distinguish between similar-sized but different shaped blades.

In the case of serrated knives, tool mark analysis of striations left on bone, cartilage [14, 15], or in soft tissues [6, 16–18] can be helpful in weapon identification. The main limitation of this method is that it is no use in case a smooth-edged blade was used, because these usually do not leave striations. Moreover, even some serrated knives might not produce detectable striations [16]. The microscopic examination of bone and cartilage injuries can give satisfactory results but cannot be used if the wound track is constituted only by soft tissues.

PMCT is a useful tool in the assessment of sharp force injuries [19, 20], but due to the limited resolution of soft tissues, the radiological visualization of the stab wound itself can be challenging [21]. Therefore, conventional autopsy provides better results than PMCT during the examination of external wounds [22]. However, filling of contrast material (CM) into the stab wound for visualization with PMCT can give better results (PMCT-CM), especially regarding stab channels [23, 24], but with some limitations. It can be used only in solid tissues because the CM will fill out the empty spaces (like lung or body cavities) thus distorting the shape of the original stab wound. The elasticity of tissues also presents a substantial limitation because it distorts the shape and size of the visualized stab channels [25] — and the enlargement of the stab wound caused by the multi-directional movement of the blade [1, 26] has a similar result. PMCT-CM is adequately accurate in assessing stab wound depth to draw conclusions about stab wound depth, but not accurate enough for identification of the blade (mean deviation is around 10%) [27]. Overall, the method can give a crude impression of the blade but cannot identify it precisely [23]. Interestingly, the results of using PMCT-CM in assessing stab wound depth are comparable with the probing method and do not give significantly better results [27]. Post-mortem magnetic-resonance imaging (MRI) has a higher sensitivity and a higher contrast resolution for soft tissues and fluids could exhibit better results in terms of wound channel depiction and measurement. With the installation of CM can also give good result when assessing the direction of the stab wound, but the radiological techniques are only poorly reproducing the features of the blade, because the CM enlarges the width of the wound cavity and leakages appear even if among muscle bands [28]. In case of stab wounds caused by weapons with blunt tip (like screwdriver), the results of post-mortem radiological methods are further limited [27].

At present, the scanning and 3D printing of objects are easily accessible, fast, cheap, and user-friendly methods and can be detected in several fields of science, including forensic sciences. An increasing number of studies deal with the potential applications for 3D printing in forensic science, affecting mostly crime scene examinations and courtroom presentations of evidence [9–11]. We presented a method for blade-wound comparison and wound track determination as another promising field of application of these methods.

Generally, 3D documentation can be achieved with two methods: either with photogrammetry whereas a computer reconstructs the 3D model from multiple images or with a special 3D scanner. The latter instruments usually project light beams onto the object. However, knife blades are particularly difficult targets: none of the abovementioned methods handles thin objects well, and also, the reflective surface can render most of the 3D scanners useless. For these reasons, we considered knives as 2D objects. The blades were scanned with a simple office flatbed scanner, which is widely available, cheap, quick, and precise way to reproduce the shape of the knife. Modelling by a photograph of the knife might be an option, but in addition to lens distortion, if the blade and the image sensor of the camera are not perfectly parallel, then perspective distortion can skew the measurements. The scanned blades were reproduced with 3D printing. This workflow can produce the plastic blade in a short period of time: scanning and editing can be completed in approximately 20 min, and printing requires approximately 60 min.

The proposed method is affordable and widely accessible, Inkscape is free and open source, and Ultimaker Cura can be downloaded and used for free as well. Following our detailed guide (Supplementary material), it is possible to produce the 3D models without any prior experience in 3D modelling. Entry level 3D printers, starting at about 250 Euros, can handle these non-demanding prints. The cost of material of printing a mid-sized knife blade is only 1–2 USD.

Ballistic gel experiment demonstrates that the replica blades could fit into the stab wounds easily, and that by installing the replica blade, it is not possible to alter the stab wound without applying large force. Also — as pork loin stabbing demonstrated — even using large force will not alter the size of the entrance wound. As dynamic stabbing measurements demonstrate, the entrance wound could be enlarged by replica blades only if an extremely large force is used, and it also results in severe bending of the knife (Fig. 5). Force measurements also show that tissue resistance rises sharply during insertion of replica knives as the borders of the original stab wound are reached, thus it is easy to avoid further damage to the stab wound (Tables 4 and 5, Figs. 6 and 7): inserting an oversized replica into the stab wound requires multiple times larger force than the stabbing with the original knife, which assures that the autopsy results are not compromised.

The results of wound assessment show that inserting 3D printed knives into the stab wounds can give adequate results in linking knives to stab wounds, even if the blade movements enlarged the wound track. Most importantly, false exclusion of knives can be avoided with a high level of probability. The method is limited in distinguishing knives with very similar blade characteristics (because these can cause almost identical injuries), but reliable if the blades differ better (Table 6).

Distinguishing superficial stab wounds (like wound “g”) also presents a limitation because only the tip of the blade enters into the tissues; therefore, the blade characteristics (shape, width) do not prevail. Inserting the various printed knives into the injuries does not alter the stab wound, and it does not modify the autopsy findings. This is supported by the fact that the last participants achieved the best results, not making any mistake in categorization. The dull plastic 3D printed knife does not create any tissue injury or leaves any striation, hence it does not compromise later tool mark analysis. Identification of the exact knife is not possible with complete certainty but excluding certain knives will decrease the number of necessary DNA examinations, hence it can lower the burden on forensic genetic laboratories.

Winkskog demonstrated that precise wound track measurement requires an object in situ [29]. Generally, metal probes are inserted into the stab wounds during forensic autopsy for wound track measurement and documentation, but only a rough estimation can be achieved with this method, since the small diameter of these probes allows relatively large movements within the wound. The presented 3D printing method presents a good solution: inserting the 3D printed knife into the injury accurately determines the stabbing direction, thus helps greatly in deciding the mechanism. While giving comparable results, the use of PMCT-CM is primarily suggested over the probing method because the latter can damage previously intact tissues [27]. Using a 3D printed knife overcomes this limitation of the probing method (which is not sharp enough to damage the tissues). The method can also be used with good results if only soft tissues are injured and overcomes the outflow of CM from the wound track in PMCT-CM.

Taking the 3D printed weapon instead of the original knife into the autopsy room rules out the possibility of cross-contamination of biological traces, thus preventing disputes in the criminal procedure.

By using the 3D printing method described above, 3D replicas of various blades can be produced to create a “library” of printed blades, which can be used to assess the possible dimensions and shape of the blade even if no suspicious knife is found during the crime scene investigation.

Scientific evidence is commonly not well understood by legal practitioners. This is especially true for jurors who usually lack experience with the interpretation of evidence [30]. The use of 3D reconstruction and 3D printing can greatly increase the understanding by the participants not educated in forensics. Blau et al. reported the highest level of understanding from the participants in a case when 3D printing was used — 93% of participants understood it “extremely well” or “well” [11].

The advantages of 3D reconstruction methods over photography in documenting and presenting injuries are already demonstrated [31, 32]. Photogrammetry allows creating 3D textured surface models from a series of overlapping photographs taken from varying viewpoints [33]. It is a less common approach for digital 3D reconstruction compared with post-mortem imaging techniques, but it is much better in documenting skin injuries [34], is easy to use, produces a high texture resolution, and is cost-effective [35, 36].

The photogrammetric 3D reconstruction of the body with the printed knife inserted to stab wound gave an unambiguous, straightforward demonstration about the stabbing direction and that the particular knife was suitable to cause the injury (see sketchfab link: https://sketchfab.com/3d-models/chest-1-11c99af60f45497db598eacfa7c9ae0a; https://sketchfab.com/3d-models/chest-2-18bdb2292d2b43eba6470146ca879491; https://sketchfab.com/3d-models/heart-and-lung-397b95c6b1474e11a32ef266c3aef672 — password for all the models: printedblade). This visualization of the stabbing helps the participants understand the procedure. Moreover, this method can also be used in undergraduate education as well as in postgraduate training.

## Conclusion

Scanning and 3D printing of knives is a useful method to identify the possible weapons in a stabbing incident without compromising the trace evidence or the autopsy results. The method is limited in distinguishing between knives of similar size and shape, but it is reliable in the exclusion of particular weapons. Insertion of the printed knife into the wound gives a good visual demonstration of the stabbing direction, thus easing the forensic reconstruction of the stabbing incident. With the combination of photogrammetry, the 3D visualization is also useful for courtroom demonstration and for educational purposes.

## Supplementary Information

Below is the link to the electronic supplementary material.

Supplementary file1 (PDF 2654 KB)
