# Supplementary material for: Application of 3D printing in assessment and demonstration of stab injuries
Source: Int J Legal Med. 2022 Jun 3;136(5):1431–42. doi: 10.1007/s00414-022-02846-6 (PMC9375752; doi:10.1007/s00414-022-02846-6)

Supplementary material of the article entitled “**Application of 3D printing in assessment and demonstration of stab injuries**” published in International Journal of Legal Medicine.

This material is intended to give a simple step-by-step guide to scan and reproduce a knife blade.

The prerequisites are the following:

- Office flatbed scanner
- A 3D printer
- Scanning utility provided by the manufacturer of the scanner
- Inkscape vector graphics editor installed (+ paths2openscad extensions)
- Ultimaker Cura slicing software installed (+ Openscad integration plugin)

Both software can be used for free. Installation steps are described at the end of this material as they need to be performed only once. In this tutorial we have used Inkscape 0.92 and Ultimaker Cura 4.11 versions, with version changes, software might change, but we are using only very basic features, I do not think any of those changes will break the workflow.

Scanning the blade

- The scanning can be performed with an office flatbed scanner (examples: Canon LiDe, HP ScanJet, Epson Perfection series). A mid-level scanner should suffice, generally the pro-level scanners are faster but do not offer higher resolution. Recommended resolution: 600 dpi.
- If DNA contamination is a concern, then prior the scanning the glass plate and the lid should be decontaminated with suitable reagent (e.g. DNA Away, Thermo-Fisher Scientific).
- The blade should be laid as flat as possible onto the glass plate. We have removed the plastic cover on the side of the scanner, which helped with positioning.
- Depending on the scanner, in certain positions, the blade migh cast a shadow and/or the edge can produce a mirror effect, what might make the tracing of the outline more difficult later. It is advised to test the optimal placement with a non-casework-knife while testing the workflow. Below the same knife was scanned with the same settings but in different orientation:

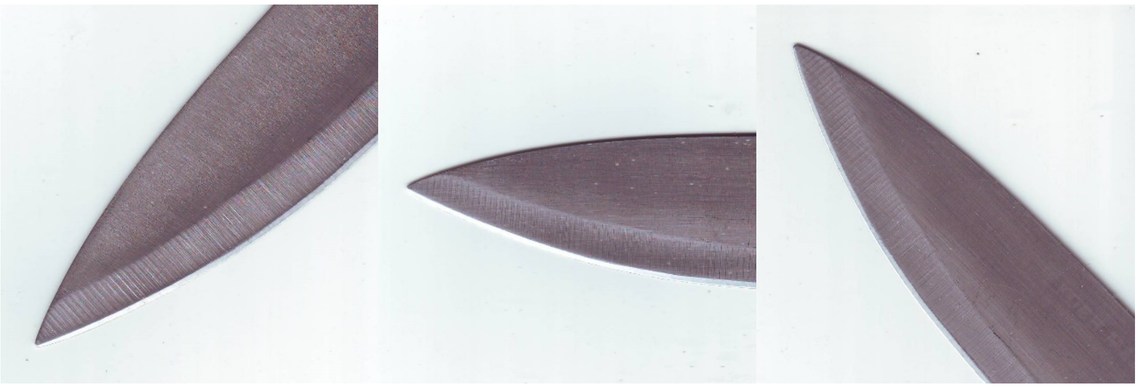

- For scaling, a known sized object (e.g. a ruler) should be also placed onto the glass plate.
- The scanned image should be similar to this:

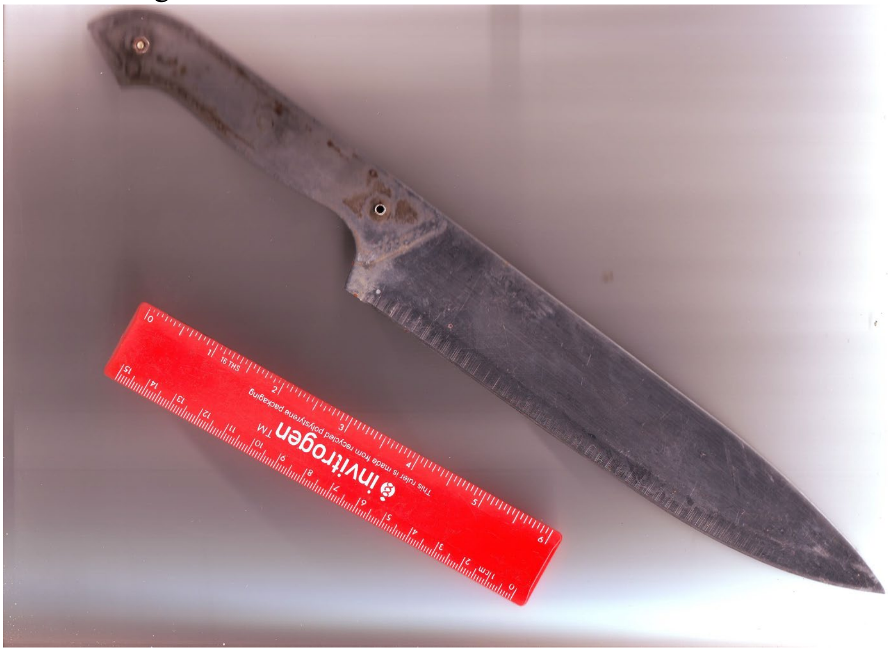

Drawing the contour in Inkscape

- Inkscape is a free and open source vector graphics editor, which can be run on Windows, Linux and MacOs systems. Open Inkscape.
- Click File/Document Properties (or Shift-CTRL-D)

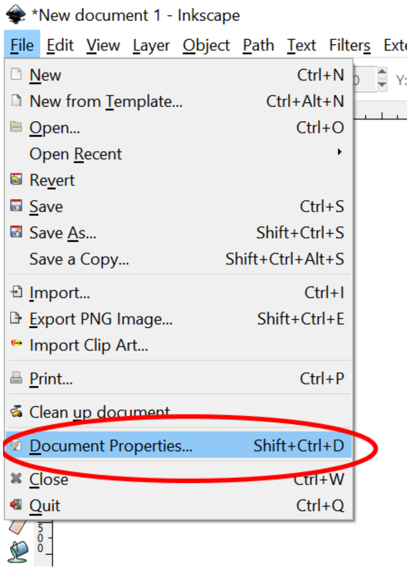

- Set both Display units and Units to px, then close the pop-up window.

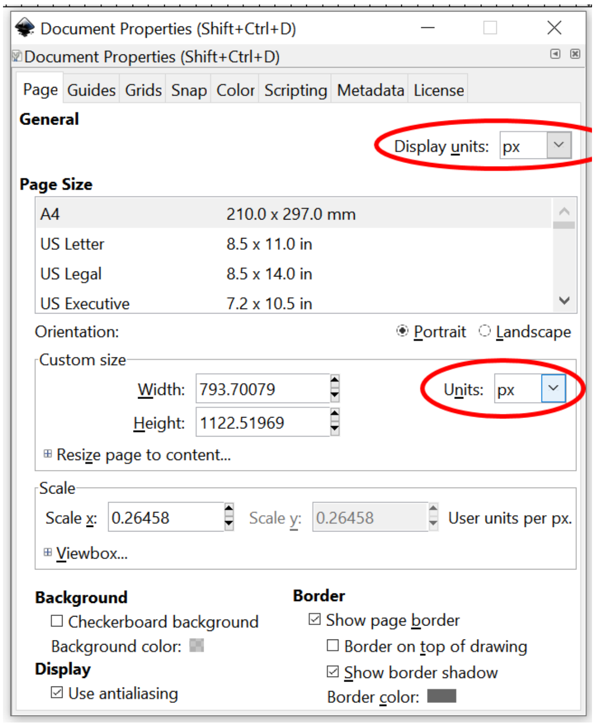

- Press File/Import (or CTRL-I) and open the image of the knife.

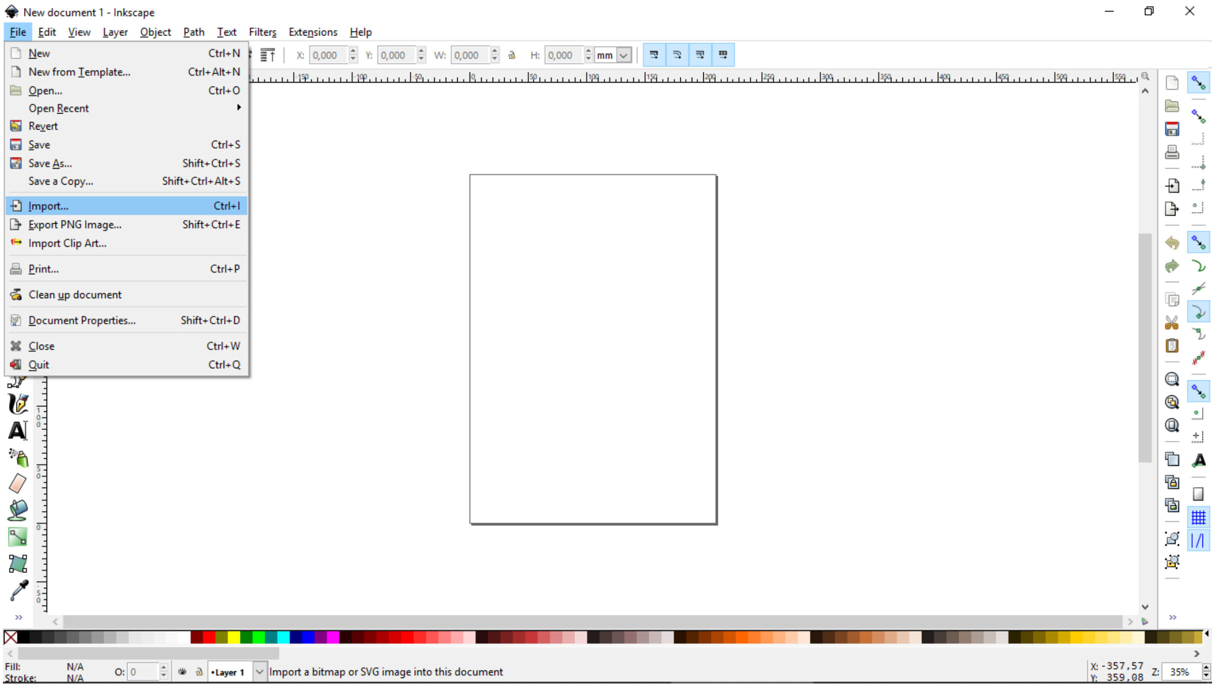

- On the left toolbar select “Draw Bezier curves” (Shift-F6).

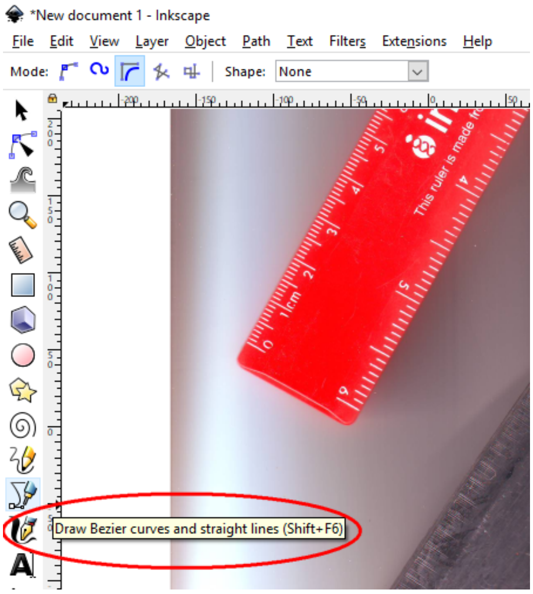

- At the top toolbar, choose “Create BSpline path” option.

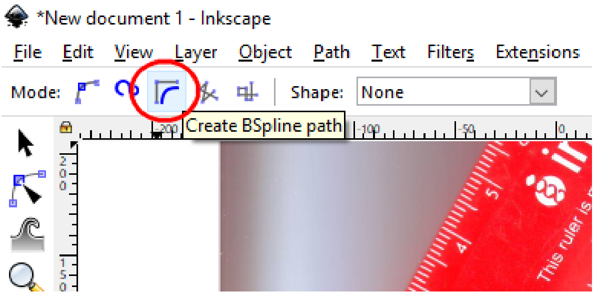

- Close up to a portion of the blade (+ or CTRL-mouse wheel) and trace the outline with the Bezier tool in small steps by placing nodes with left mouse button. The view can be moved by holding down the middle mouse button and moving the mouse.
- The starting point and the end point should be the same point, so the path will be closed.
- If necessary, the path can be edited after clicking "Edit path by nodes" on the left hand toolbar (F2). Then the individual nodes on the path can be moved or erased.

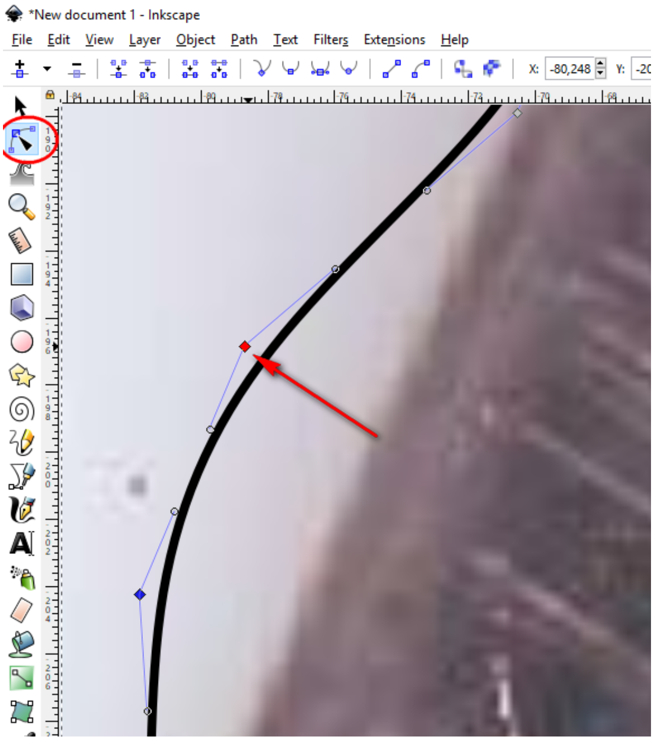

- For size scaling, draw a rectangle between two markers of the scanned ruler. Use the Bezier tool, but this time choose the "Create a sequence of straight line segments" option from the top toolbar. Make sure, that the first and last points are joined.

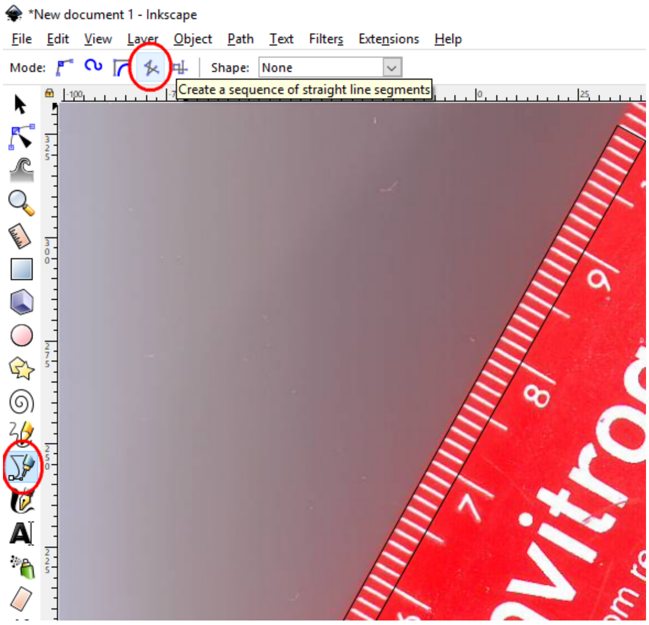

- When finished the tracing, click on the path to choose it. Then click Extensions/Generate from path/Paths to OpenSCAD.

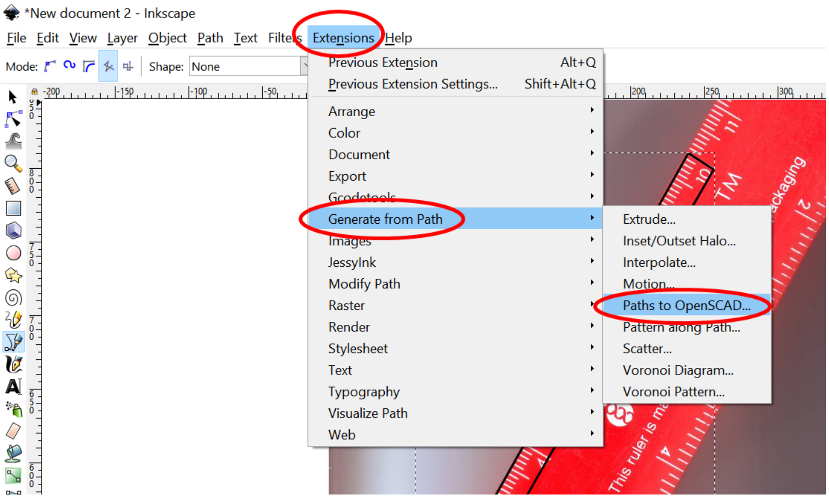

- Set the following parameters: Output file: the filename to save the model (file extension should be .scad) Height: the height of the model in mm; Smoothing: set to 0. Click apply. The file will be saved to the Documents folder.

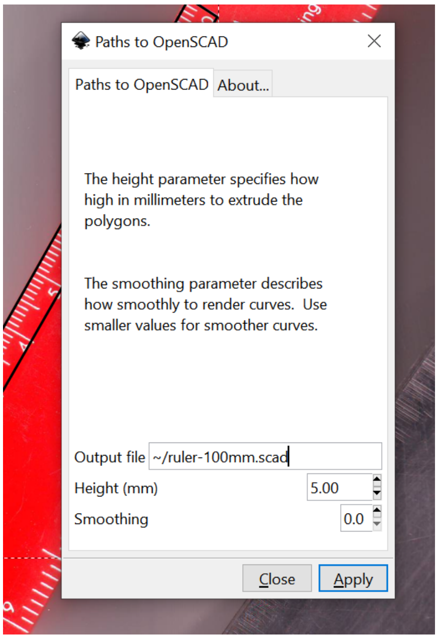

- Repeat the same steps with the track of the knife. Set the height the measured thickness of the blade.

Slicing the model with Ultimaker Cura

- Open Ultimaker Cura, and drag and drop the ruler.scad file.
- Check the size of the ruler.

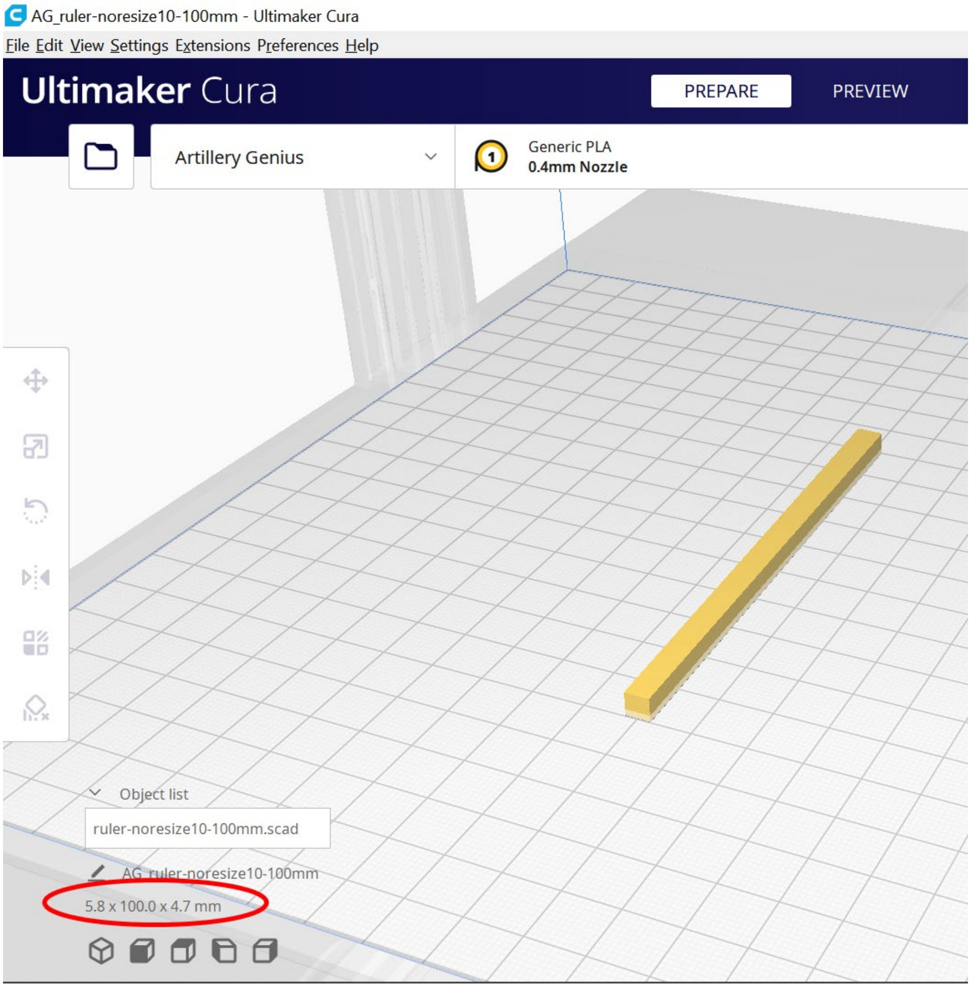

- Fine tune the scaling if necessary.

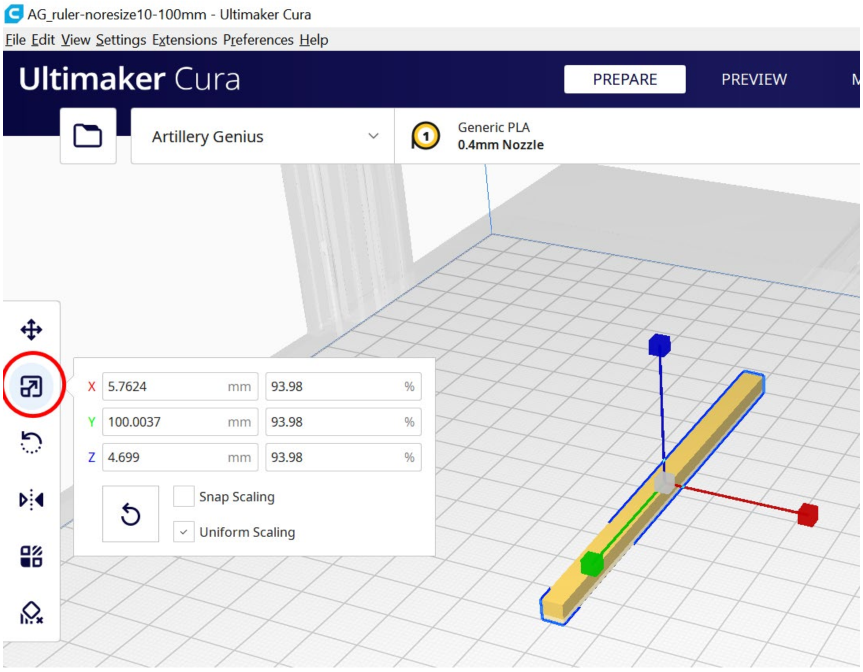

- Import the .scad file of the blade. Scale it if necessary. Slice and print with the preferred settings.

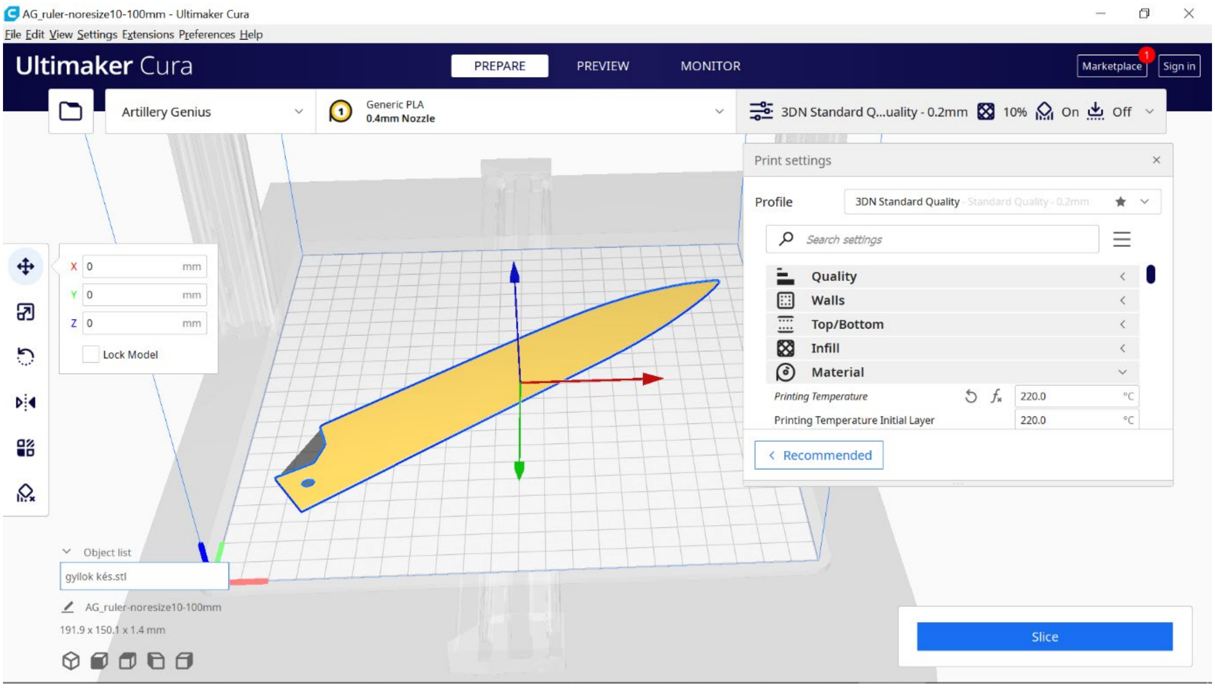

Installation steps

Installing Inkscape

- Visit inkscape.org, download and install the current version of the software.

Installing Inskcape extension

- Visit https://www.thingiverse.com/thing:25036/files, and download paths2openscad-6.zip
- Unzip the archive
- On Windows copy the unzipped files into C:/Program Files/Inkscape/share/extensions folder.
- On Linux or MacOs, copy those file into ~/.config/inkscape/extensions/
- Close and restart Inkscape if it was running.

Installing Ultimaker Cura

- Visit https://ultimaker.com/software/ultimaker-cura Download and install the current version of the software.

Installing Ultimaker Cura plugin

- Open Cura, click on Marketplace on the top right corner.

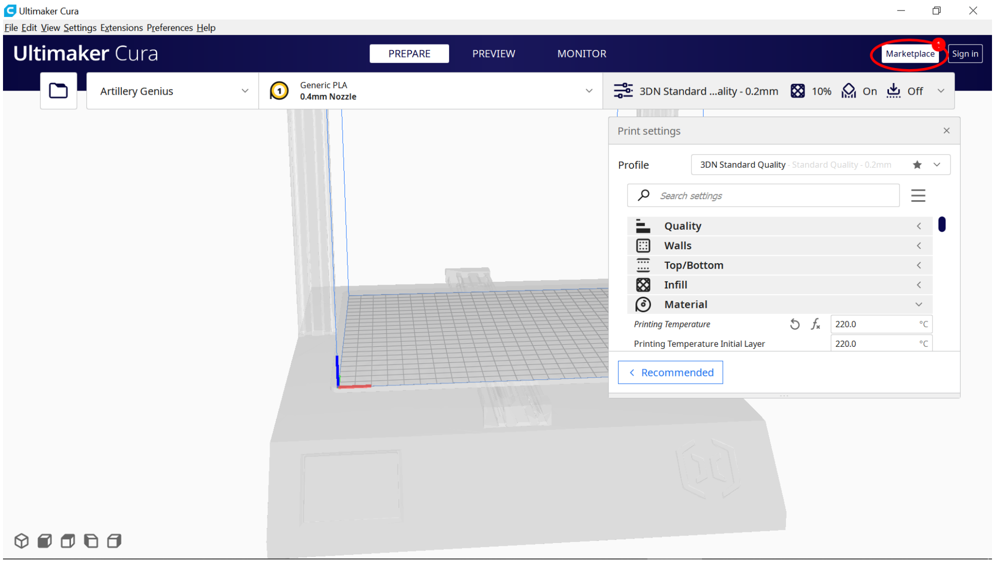

- Choose “OpenSCAD integration” from the plugins list.

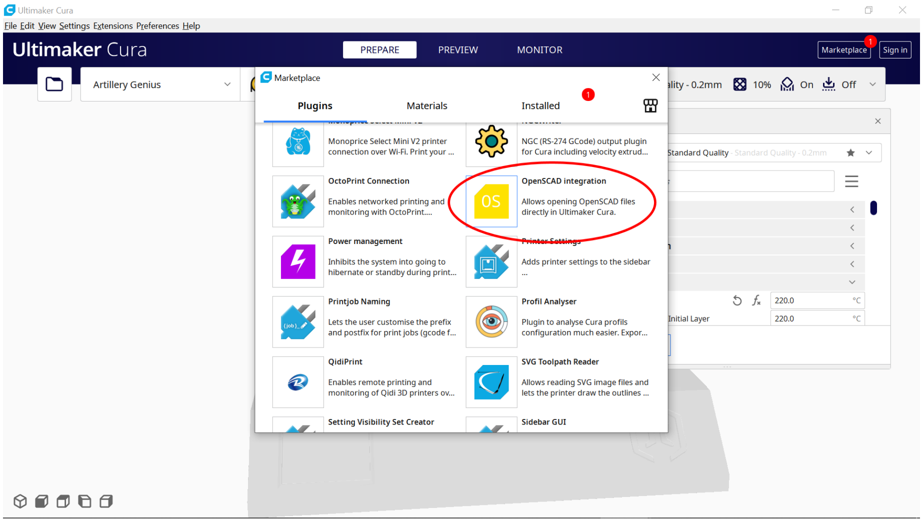

- Install the plugin.

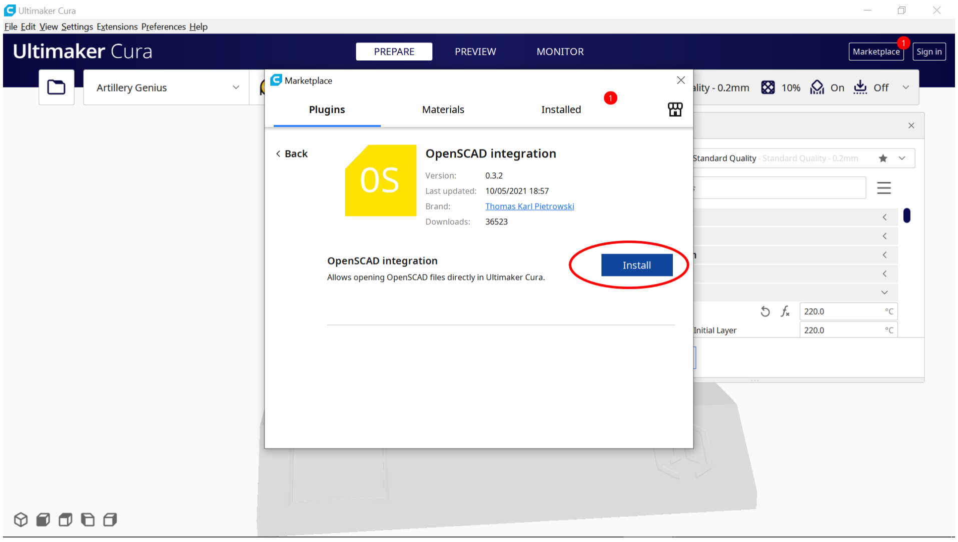

Supplement: Supplementary file 1 — Supplementary file1 (PDF 2654 KB) [file 414_2022_2846_MOESM1_ESM.pdf]
